# Watercress oil loaded with gel: evaluation of hemolysis inhibition, antioxidant, antimicrobial, and healing properties

**DOI:** 10.3389/fphar.2024.1424369

**Published:** 2024-08-23

**Authors:** Bandar Alharbi, Husam Qanash, Majed N. Almashjary, Heba Barnawi, Abdu Aldarhami, Ghaida Alsaif, Fahad Alsamaan, Mohammad Khalil Monjed, Humood Al Shmrany, Abdulrahman S. Bazaid

**Affiliations:** ^1^ Department of Medical Laboratory Science, College of Applied Medical Sciences, University of Ha’il, Hail, Saudi Arabia; ^2^ Medical and Diagnostic Research Center, University of Ha’il, Hail, Saudi Arabia; ^3^ Department of Medical Laboratory Sciences, Faculty of Applied Medical Sciences, King Abdulaziz University, Jeddah, Saudi Arabia; ^4^ Hematology Research Unit, King Fahd Medical Research Center, King Abdulaziz University, Jeddah, Saudi Arabia; ^5^ Department of Medical Microbiology, Faculty of Medicine, Al Qunfudah, Umm Al-Qura University, Makkah, Saudi Arabia; ^6^ Department of Biology, College of Science, Umm Al-Qura University, Makkah, Saudi Arabia; ^7^ Department of Medical Laboratory Sciences, College of Applied medical sciences, Prince Sattam bin Abdulaziz University, Alkharj, Saudi Arabia

**Keywords:** vaseline gel, watercress oil, antioxidant, anti-hemolysis, antimicrobial

## Abstract

Plant-derived compounds are renowned for their remarkable pharmacological properties, holding immense promise for therapeutic interventions in human health. In this study, we aimed to assess the antimicrobial, anti-hemolytic, antioxidant, and wound healing attributes of watercress oil incorporated into Vaseline gel (OLG) compared to watercress oil alone. OLG was formulated through a meticulous process involving the addition of Vaseline gel to the oil under agitation conditions. High-performance liquid chromatography analysis of watercress oil unveiled a rich array of phenolic compounds, including gallic acid (10.18 μg/mL), daidzein (3.46 μg/mL), and hesperetin (3.28 μg/mL). The inhibitory zones caused by watercress oil alone against a spectrum of pathogens, including *Staphylococcus aureus, Escherichia coli, Klebsiella pneumoniae, Enterococcus faecalis,* and *Candida albicans*, were measured at 25 ± 0.3, 26 ± 0.1, 22 ± 0.2, 25 ± 0.2, and 24 ± 0.1 mm, respectively. Notably, OLG exhibited slightly larger zones of inhibition (27 ± 0.2, 30 ± 0.2, 24 ± 0.1, 28 ± 0.1, and 25 ± 0.3 mm) against the same microbial strains. Furthermore, the minimum inhibitory concentration (MIC) of OLG against *E. coli* and *E. faecalis* was lower compared to watercress oil alone, indicating enhanced efficacy. Similarly, the minimum bactericidal concentration (MBC) of OLG was notably lower across all tested bacteria compared to watercress oil alone. Inhibition of bacterial hemolysis, particularly *K. pneumoniae*, was significantly enhanced with OLG treatment, showcasing reductions of 19.4%, 11.6%, and 6.8% at 25%, 50%, and 75% MIC concentrations, respectively, compared to watercress oil alone. The antioxidant activity of both oil and OLG was quantified with IC50 values of 2.56 and 3.02 μg/mL, respectively. Moreover, OLG demonstrated remarkable efficacy in wound healing assays, with notable enhancements in migration rate, wound closure, and area difference compared to control cells. In light of the observed antibacterial, antifungal, anti-hemolytic, and wound healing properties of OLG, this formulation holds therapeutic potential in treating microbial infections and promoting wound healing.

## 1 Introduction

The utilization of natural products, especially of plant origin has been shown to offer great benefits to human health and the food industry, serving as safe alternatives to synthetic chemical compounds. Therefore, the field of discovery, development and evaluation of these natural products has recently gained serious attention due to the promising potential therapeutic use (Al-Rajhi et al., 2022; Yahya et al., 2022; Al-Rajhi et al., 2023; Alsalamah et al., 2023). In the current decade, *Nasturtium officinale* (watercress) is being evaluated as a promising herb with excellent pharmaceutical value. Numerous investigations have provided valuable information about the medicinal potential within different parts of this plant, which has led to the recommendation of its promising utilization in the pharmaceutical industry. In the English language, this plant is linked with different common names, such as garden cress, salad rocket, arugula, and rocket. Similarly, it is also known as salatrauke, eruca, roquette, rucola, and garger in German, Spanish, French, Italian, and Arabic, respectively. Watercress contains significant amounts of vitamins, pigments, and minerals, including riboflavin, iron, calcium, phosphorus, manganese, as well as vitamins A, B6, and C (Klimek-Szczykutowicz et al., 2018). Abdulkareem and Aldhaher have recently documented that watercress oil would be a promising natural component with abundant nutritional and therapeutic values (Abdul Kareem and Al Dhaher, 2021).

Watercress is a vital medicinal plant, primarily used in traditional medicine (Teixidor-Toneu et al., 2016), and the extracted oil (watercress oil) was reported with broad biological activities, including anti-tuberculosis (Halberstein, 2005), broad spectrum antibacterial activities (Bahramikia and Yazdanparast, 2010), exerting cardioprotective actions (Pandey and Watercress, 2018), and antioxidant properties (Zeb, 2015; Ramezani et al., 2021). Multiple studies have reported the broad antibacterial and antifungal activities of watercress oil (Qanash et al., 2023a). According to the study conducted by Alagawany et al., significant inhibitory effects were monitored towards Enterobacteriaceae, and coliforms counts using watercress oil (Alagawany et al., 2018). Moreover, larger inhibition zones were observed with the combined application of watercress oil with the calcium hydroxide paste than those designed by each tested sample separately. The healing, anti-inflammatory, and antimicrobial properties of watercress oil, which promoted epithelialization from wound peripheries, were documented (Zinadah, 2008). The effectiveness of watercress oil in wound healing was investigated in comparison with traditional antibiotics and untreated group (control), in which the closure time and contracting ability in both the watercress oil and antibiotic groups were significantly better from those of the untreated group of rabbits (Zinadah, 2008).

The use of gels for the topical application of plant extracts and their essential oils has been explored, with Carbopol 940 showing promise as a gelling agent in essential oil formulations with demonstrated antifungal and antibacterial activities (Ullah et al., 2023; Pandey et al., 2010). Several studies have highlighted the incorporation of numerous essential oils into gelatin coatings, edible films, and other biopolymers for various purposes, including packaging, flavoring, and product preservation (Acevedo-Fani et al., 2015; Atarés and Chiralt, 2016; Goudoulas et al., 2022; Monfared-Hajishirkiaee et al., 2023). A study reported that microencapsulated watercress oil with whey protein reduced the oil oxidation in the cookies (Umesha et al., 2015). While pullulan coated with the extract of watercress was found to increase their antioxidant potential, delaying chemical degradation, and extending the fish fillets shelf-life (Shahhoseini et al., 2021). Vaseline has been part of skincare routines, keeping skin moist during post-surgery healing, besides it will form a protective barrier that help to protect certain parts of human body from constant exposure to moisture. In addition, Vaseline gel can be used as a carrier for watercress oil to enhance the activity of oil, limiting the releasing of active constituents and keeping the oil from oxidation. Therefore, the current investigation aims to evaluate the antimicrobial and antioxidant activities and wound healing properties of watercress oil and compare it with that resulted from watercress oil loaded into Vaseline Gel. The viscosity caused by gel offers information on the flow behavior of semi-liquid formulations. The specific properties of each type of gel base can be enhanced in the presence of various substances in the composition; therefore the ability of both watercress oil alone and watercress oil incorporated with Vaseline Gel to reduce/inhibit the hemolysis effect caused by pathogenic bacteria will be assessed.

## 2 Materials and methods

### 2.1 Watercress oil and chemical materials

Watercress (*N. officinale*) oil was purchased from local market (Hail, Saudi Arabia). Dimethyl-sulfoxide (DMSO), Tween 80, Mueller-Hinton agar, Potato dextrose agar and other applied chemicals in the current study were purchased from Merck (St. Louis, MO, United States).

### 2.2 Phenolic contents analysis of watercress oil via high-performance liquid chromatography (HPLC)

In the analytical process, 5 μL of watercress oil, extracted using methanol, was injected into High-performance liquid chromatography system. Specifically, an Agilent 1,260 series with an Eclipse C18 column (4.6 mm × 250 mm, 5 m) was utilized for content separation. The mobile phase was composed of water (A) and 0.05% trifluoroacetic acid in acetonitrile (B), with a flow rate of 0.9 mL/min. The gradient linear was automated into the mobile phase, following this sequence: 82% A at 0 min, 80% A at 0–5 min, 60% A at 5–8 min, 12% A at 8 min, 15% A at 12 min, 16% A at 15 min, and 20% A at 16 min. A wavelength detector set at 280 nm was used, and the column was maintained at 40°C throughout the entire process. The identification and quantification of phenolic compounds were performed qualitatively and quantitatively based on injected standards (supplement) in the HPLC system (Abdelghany et al., 2021).

### 2.3 Preparation of oil-loaded vaseline gel (OLG)

The Vaseline gel was slowly added to oil under agitation conditions, incorporating Tween 20 (2 mL) to achieve a homogenized mixture. Additionally, a Vaseline gel without oil was prepared using the same above procedure and utilized as a control in certain experiments.

### 2.4 Agar well diffusion and microdilution assay

The antimicrobial activities of oil and OLG were evaluated using the agar well diffusion technique. Fresh active cultures of bacteria and fungi, including *Staphylococcus aureus* (ATCC 6538), *Escherichia coli* (ATCC 8739), *Klebsiella pneumoniae* (ATCC 13883), *Enterococcus faecalis* (ATCC 10541), *Candida albicans* (ATCC 10221), and *Mucor circinelloides* (AUMMC 11656), were utilized. Theses microbes was selected as they are commonly when screen for antimicrobial activity, so they are representative for common pathogenic bacteria of Gram positive and negative as well as both unicellular and multicellular fungi. In addition, selected bacteria are commonly reported with drug resistance, and it is a very desirable feature for a potential antimicrobial compound to show activity towards pathogenic bacteria/microbes that are associated with drug resistance These cultures were grown for 24 h (bacteria) or 72 h (fungi). To conduct the experiment, 10 µL of bacterial or fungal suspension containing 1.5 × 10^8^ CFU/mL or 1.5 × 10^6^ CFU/mL, respectively, were streaked on Muller Hinton Agar for bacteria or Potato dextrose agar for fungi. Subsequently, agar plugs were removed using a sterile cork borer (6 mm). Under sterile conditions, wells were loaded with 25 µL of oil, OLG, standard antimicrobials (Gentamicin for bacteria and clotrimazole for fungi as positive controls) or DMSO (10% as negative control). The inoculated plates with bacteria or fungi were then incubated at 37°C or 25°C for 24 h or 72 h, depending on the optimum growth requirements of microorganism under examination. At the end of the incubation period, the diameters of the clear zones that formed around the wells were measured as an indication of antimicrobial activity (Abdelghany, 2013).

The minimum inhibitory concentration (MIC) of oil and OLG was determined using the microdilution assay. A 12-hour-old microbial inoculum was prepared, and microbial suspensions were adjusted to 0.5 McFarland standard turbidity. The oil and OLG were dissolved in 10% DMSO and then diluted to achieve various concentrations up to 1,000 μg/mL (Stock solution (1,000 μg/mL)was serial diluted to obtains different concentrations). Each concentration was added to sterile tubes containing 10 mL of Mueller-Hinton broth medium. In 96-well plates, 95 µL of Mueller-Hinton broth medium and the microbial suspension (1.5 × 10^8^ CFU/mL of bacteria/yeast) were dispensed into each well. Additionally, inoculated Mueller-Hinton broth with 10% DMSO was used as a negative control. The plates were then incubated for 24 h at 37°C. The MIC was determined based on the lowest concentration of the oil or OLG that inhibited microbial growth. The minimum bactericidal concentration (MBC) was defined as the lowest quantity of oil or OLG that completely killed the microorganisms. To determine the MBC, 20 μL of broth from each well that displayed no microbial growth was cultivated onto Muller Hinton agar and incubated for 24 h at 37°C. After incubation, the microbial growth was visualized, and MBC was recorded (French, 2006).

### 2.5 Hemolysin inhibition assay

The hemolysis inhibition assay, as described by Rossignol et al. (Barnawi et al., 2023), was conducted to determine the anti-hemolytic activities of oil and OLG towards produced hemolysis from tested bacteria. Sub-MIC concentrations, including 25%, 50%, and 75% of the MIC of oil and OLG, were used to treat cultures of *K. pneumoniae*, *S. aureus*, *E. coli*, and *E. faecalis*, along with untreated cultures of the same bacteria as controls (untreated). Tested bacteria treated with 25%, 50%, and 75% of MIC (sub-MIC) or untreated cultures were adjusted to an OD 600 of 0.4 and then centrifuged at 21,000× g for 20 min. The collected supernatant (500 µL) was added to a 2% fresh erythrocyte suspension in 0.8 mL saline, followed by an incubation period at 37 °C for 2 h. Afterward, the suspension was centrifuged for 10 min at 11,000× g and 4 °C. A 0.1% solution of Sodium Dodecyl Sulfate (SDS) was added to the erythrocyte suspension as a positive control for complete hemolysis, while negative controls (un-hemolyzed erythrocytes) made of erythrocytes dissolve in Luria Broth followed the same incubation conditions. The released hemoglobin was quantified by measuring the absorbance at 540 nm. The level of hemolysis in the sub-MIC treated cultures was presented as a mean ± standard error of the percentage change from the hemolysis of untreated control cultures. The hemolysis inhibition percentage was calculated using an equation and the released hemoglobin was compared with the positive and negative controls to determine the extent of hemolysis inhibition (Equation 1).

$$\text{Hemolysis inhibition}\left(\%\right)=\frac{\text{Sample with bacterial culture}\left(\text{untreated}\right)-\;\text{Negative control}\;}{\text{Positive}\;cont–rol\;-\;\text{Negative control}}\times 100$$ (1)

### 2.6 Antioxidant activity

Oil and OLG have been tested for their antioxidant potential via DPPH (1,1-diphenyl-2-picryl hydroxyl) radical scavenging assessment. About 3 mL of dis-solved Oil and OLG in ethanol at various concentrations (3.9, 7.8, 15.62, 31.25, 62.5, 125, 250, 500, and 1,000 μg/mL) were mixed with 1 mL of a 0.1 mM DPPH solution (in ethanol). The reaction mixture was shaken for 25 min at room temperature (25 °C). Subsequently, the reaction mixture absorbance was recorded at 517 nm via UV-VIS Milton Roy Spectrophotometer. The amount of Oil and OLG necessary to obstruct 50% of the DPPH free radical was documented using a log concentration of inhibition curve (IC_50_) (Qanash, 2023b). All findings of the antioxidant potential were compared to the standard antioxidant agent (ascorbic acid) (Abdel Ghany et al., 2019; Rajamanickam et al., 2018).

### 2.7 Wound healing via scratch wound closure assay

The examination of the scratch wound was done using a multiwell plate. A 10 μg/mL fibronectin extracellular matrix substrate was applied to the plate, and subsequently a 2-h incubation period at 37 °C. Following the release of the unbound extracellular matrix, phosphate-buffered saline was used as a wash. Trypsin was used to separate the developing cells from a dish containing tissue culture. On the scratch wound assay plate, the cells were grown, and then they were incubated to allow the cells to proliferate and form a confluent monolayer. Using a pipette tip, the monolayer cell—including the confluent monolayer was scraped. After scratching, gently wash the cell monolayer to get rid of any separated cells. Subsequently, swap it out for a new medium that contains rosemary extract. For 24–48 h, the plate was preserved at 37 °C in the incubator of cell culture. Phosphate-buffered saline was applied to wash the cell monolayer following the incubation period. The cells were then fixed with 3.7% paraformaldehyde for 15 min. For 10 minutes, the cells were stained with crystal violet (1% in ethanol). Next, a phase-contrast microscope was used to look at the cell culture (French, 2006). The following calculation was recorded based on the following formula:

$$\text{Rate of migration }\left(\text{RM}\right)=\frac{\text{Wi}\;–\;\text{WF}}{T}\times 100$$

WI, meaning the width (µm) of initial wound; WF, meaning the wound width (µm) of final wound; T, meaning the time span of the test in hours

$$\text{Wound closuer }\%=\frac{\text{At}0\;–\;\text{At}∆t\;}{\text{At}=0}\times 100$$

Where, *A*_*t0*_ *meaning the area of intial wound; A*_*t∆t*_ *meaning the area of wound after n hours*

Area difference %=intial area− final are

### 2.8 Statistical analysis

The antimicrobial activity results were presented as mean ± standard deviation (SD) based on triplicate experiments. Student t-test was used to calculate significance (*p* < 0.05) difference of compared to control. Data analysis was conducted using Microsoft Excel, Origin Pro (Version 9), and GraphPad Prism software (Version 8) to process and interpret the experimental findings.

## 3 Results and discussion

**FIGURE 1:**
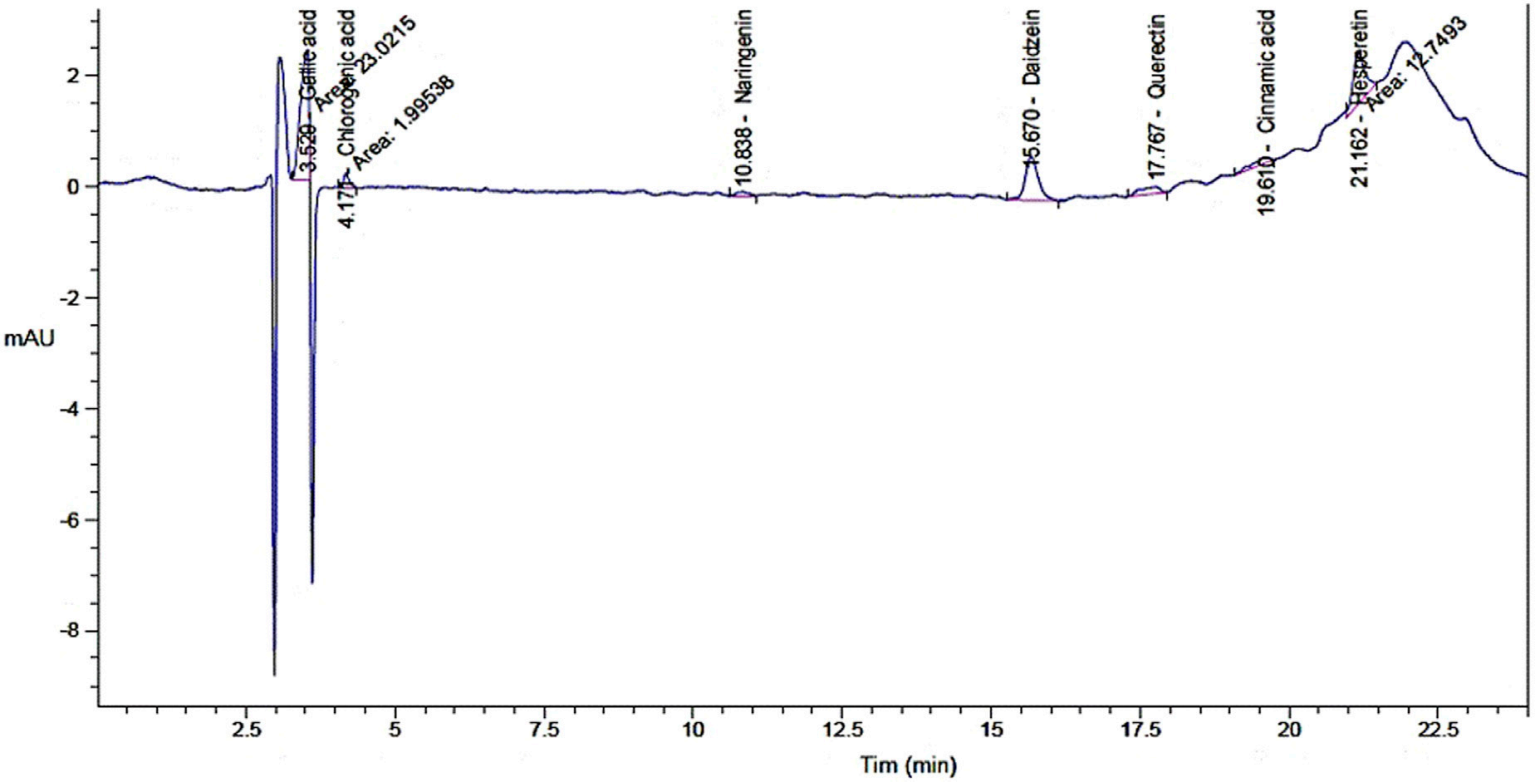
Chromatogram analysis for phenolic constituents of watercress oil by High-performance liquid chromatography (HPLC).

The current evaluated the antimicrobial and antioxidant activities and wound healing properties of watercress oil alone and watercress oil loaded into Vaseline Gel. In addition, the ability of both compounds to reduce/inhibit the hemolysis effect caused by pathologic bacteria was assessed. The analysis of watercress oil revealed the presence of various compounds, including gallic acid, daidzein, hesperetin, quercetin, chlorogenic acid, naringenin, and cinnamic acid, with concentrations of 10.18, 3.46, 3.28, 1.93, 1.35, 0.54, and 0.21 μg/mL, respectively (Table 1; Figure 1). However, certain compounds, such as catechin, caffeic acid, rutin, and kaempferol were not detected in the watercress oil, as indicated in the analysis using injected standard compounds in the HPLC (Table 1). It is worth noting that the detected compound gallic acid has previously exhibited antibacterial activity against *Pasteurella multocida* and *Mannheimia haemolytica*, which are causative agents of Bovine respiratory disease (Al bratty et al., 2021). Similarly, chlorogenic acid has demonstrated antimicrobial activity in a recent investigation (Alsalamah et al., 2023).

**TABLE 1:** Identified phenolic constituents in watercress oil by HPLC.

Component	Retention time	Area	Area (%)	Concentration (µg/mL)
Gallic acid	3.53	23.02	40.99	10.18
Chlorogenic acid	4.17	2.00	3.55	1.35
Naringenin	10.84	1.14	2.03	0.54
Daidzein	15.67	11.95	0.00	3.46
Quercetin	17.77	3.04	5.42	1.93
Cinnamic acid	19.61	2.25	4.00	0.21
Hesperetin	21.16	12.75	22.70	3.28

Despite the limited detection of phenolic compounds in watercress oil through the HPLC, this does not preclude the presence of other biologically active constituents. Lack of detected compounds could be linked with the used extraction methodology or instrumentation as it could be not appropriate for the yield of associated compounds. Another analysis, utilizing GC–MS, has recently identified thirty-eight chemical constituents in watercress oil, including glycosides, aliphatic hydrocarbons, sulfur compounds, terpenoids, and silicon compounds [34]. Furthermore, the GC–MS analysis has provided insights into the fatty acid profile of watercress oil, revealing the presence of various fatty acids, such as palmitic acid, behenic alcohol, cis-vaccenic acid, oleic acid, gondoic acid, paullinic acid, and erucic acid (Alghonaim et al., 2024).

**FIGURE 2:**
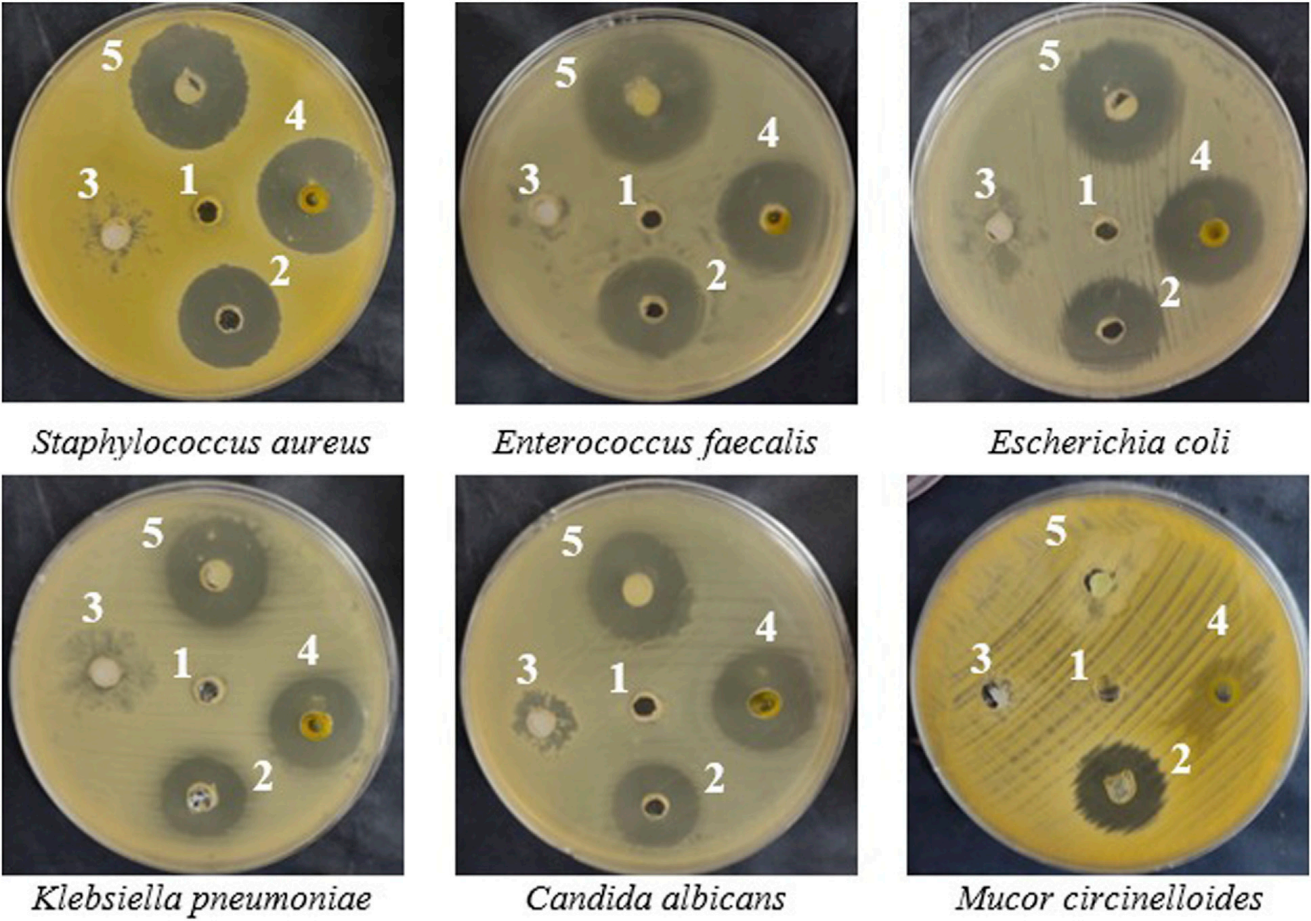
*In-vitro* inhibition (inhibitory zones) of microbial growth caused by negative control dimethyl sulfoxide (DMSO) (1), positive control (Gentamicin for bacteria and clotrimazole for fungi) (2), Vaseline gel (3), watercress oil (4), and watercress oil loaded with Vaseline gel (OLG) (5).

The incorporation of watercress oil into the gel has led to stronger antimicrobial activities against all tested microorganisms (Table 2; Figure 2). Size of inhibition zones caused by oil loaded with gel were 27 ± 0.2, 30 ± 0.2, 24 ± 0.1, 28 ± 0.1, and 25 ± 0.3 mm, whereas reduced zone sizes (25 ± 0.3, 26 ± 0.1, 22 ± 0.2, 25 ± 0.2, and 24 ± 0.1 mm) were observed for the oil alone against *S. aureus*, *E. coli*, *K. pneumoniae*, *E. faecalis*, and *C. albicans*. Notably, the Vaseline gel alone exhibited lower inhibition zones against *S. aureus*, *E. faecalis*, and *C. albicans*, while displaying no antimicrobial activity against *E. coli* and *K. pneumoniae*. The increased activity (large zones) of OLG construction was observed, which might be due to the increased diffusion/penetration of the oil into the tested bacteria. This is because water content of vaseline gel was reported to enhance diffusion/penetration of oils through the polar peptidoglycan layer within the cell wall of bacteria. In addition, vaseline gel has improved the stability of tested oil. Thus, enhanced diffusion/penetration as well as better stability of the tested oil will definitely lead to larger zone of inhibition (stronger activity). It is also worth mentioning that the filamentous fungus *M. circinelloides* was not inhibited by watercress oil, Vaseline gel, or the oil loaded gel (OLG). All results of inhibition zones using tested samples were compared to the inhibition zones caused by common antibiotics (Gentamycin) and antifungal (clotrimazole) as positive control (Table 2).

**TABLE 2:** Antimicrobial activity of Vaseline gel, watercress oil, watercress oil loaded with Vaseline gel (OLG) and positive controls (Gentamicin for bacteria and clotrimazole for fungi).

Tested microorganisms	Inhibition zone (mm)			
	Vaseline	Oil	OLG	Control
*Staphylococcus aureus*	10 ± 0.1	25 ± 0.3	27* ± 0.2	23 ± 0.3
*Escherichia coli*	NA	26 ± 0.1	30* ± 0.2	24 ± 0.1
*Klebsiella pneumoniae*	NA	22 ± 0.2	24* ± 0.1	19 ± 0.2
*Enterococcus faecalis*	11 ± 0.1	25 ± 0.2	28* ± 0.1	24 ± 0.1
*Candida albicans*	14 ± 0.2	24* ± 0.1	25* ± 0.3	20 ± 0.1
*Mucor circinelloides*	NA	NA	NA	17 ± 0.2

Furthermore, the oil loaded gel (OLG) demonstrated a low minimum inhibitory concentration (MIC) against tested microorganisms, including 7.8 μg/mL for *E. coli* and 15.62 μg/mL for *E. faecalis*, compared to watercress oil alone. Similar MIC values were recorded against *S. aureus* and *K. pneumoniae*. This means lower concentration (lower MIC values) of the OLG is required to inhibit the tested microbe when compared to the other oil without gel as higher MIC value is required. Additionally, the minimum bactericidal concentration (MBC) value of OLG was lower than that of watercress oil against all tested bacteria, with equal values observed against *C. albicans*. The stronger activity of OLG compared to watercress oil alone might be due to a higher yield/concentration of the active compound from the extract watercress oil was achieved when using the gel. Moreover, the bactericidal characteristic was attributed to watercress oil and OLG due to the MBC/MIC index being less than 4 (Table 3). According to French (French, 2006), an MBC/MIC ratio of less than four times the MIC indicates bactericidal characteristics of the tested samples.

**TABLE 3:** Minimum inhibitory concentration (MIC), Minimum bactericidal concentration (MBC), and MBC/MIC index of Vaseline gel, watercress oil, and watercress oil loaded with Vaseline gel (OLG).

Tested microorganisms	MIC (µg/mL)			MBC (µg/mL)		MBC/MIC index	
	Vaseline	Oil	OLG	Oil	OLG	Oil	OLG
*Staphylococcus aureus*	-	15.62	15.62	31.25	15.62	2	1
*Escherichia coli*	-	15.62	7.8	31.25	15.62	2	2
*Klebsiella pneumoniae*	-	62.5	62.5	125	62.5	2	1
*Enterococcus faecalis*	-	31.25	15.62	62.5	15.62	2	1
*Candida albicans*	1,000	62.5	62.5	125	125	2	2

*Significant difference compared to control.

Several antibacterial mechanisms have been associated with the constituents of watercress oil. For instance, the inhibition of extracellular enzymes, interruption of active transport, and electron flow were attributed to tannins; while inhibition of cytoplasmic membrane function, dysfunction of nucleic acid synthesis, and depletion of energy metabolism were attributed to flavonoids. Kaur et al. noticed that cinnamon oil, when incorporated in topical gels, exhibited better antibacterial activity than bulk oil (Kaur et al., 2021). Additionally, the extracted lavender and thymus from the essential oils of *Lavandula angustifolia* and *Thymus vulgaris*, respectively, were applied in the form of massage using Vaseline and reflected strong inhibitory action toward *Streptococcus* and *Staphylococcus* species (Abboud et al., 2015).

**FIGURE 3:**
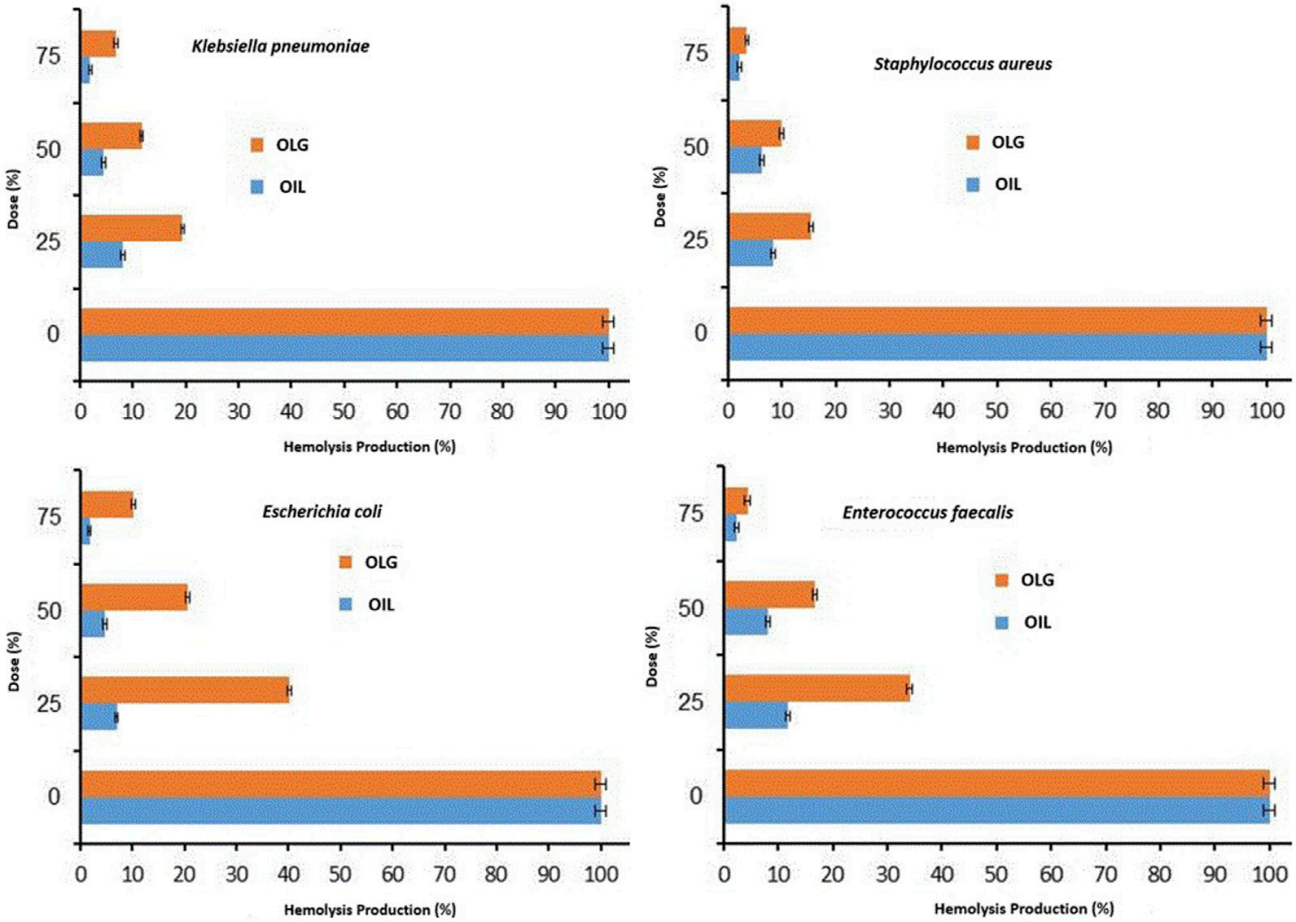
Inhibition rate of multiple Gram-positive and negative bacterial hemolytic activities at different doses/concentrations (0% MIC, 25% MIC, 50% MIC, and 75% MIC) of watercress oil (Oil) and watercress oil loaded with Vaseline gel (OLG). Error bars in the Figure represent the calculated standard deviation (SD).

**FIGURE 4:**
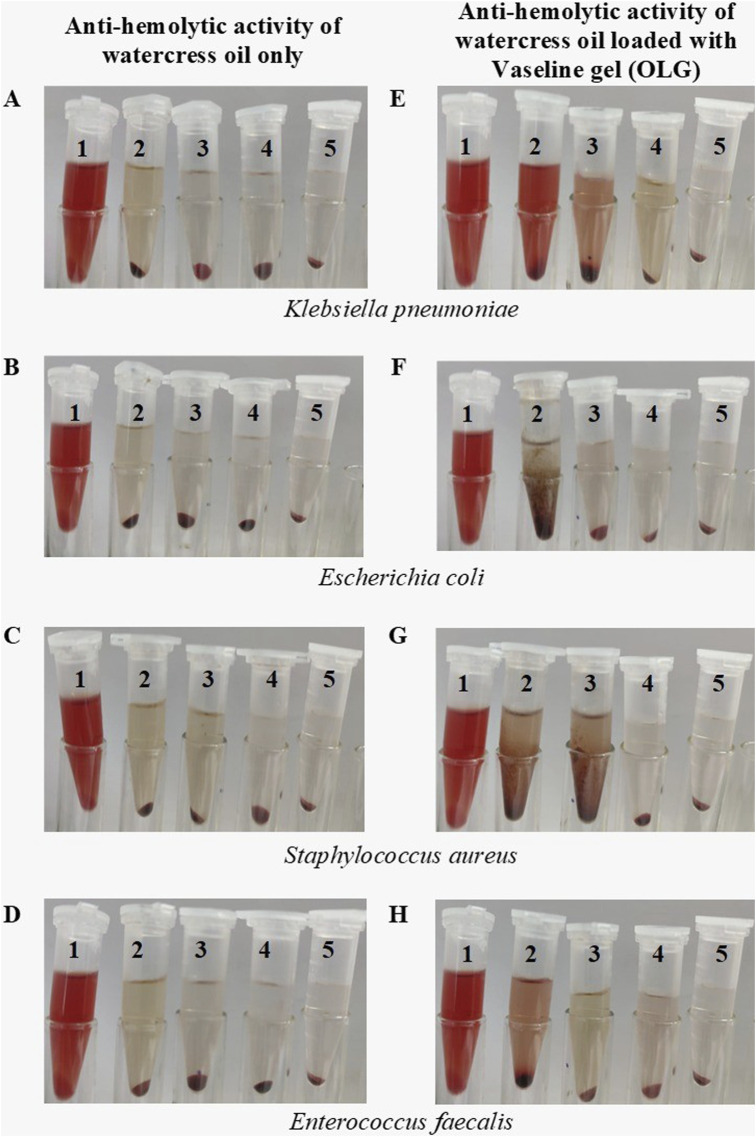
Visual observation of multiple bacterial hemolytic activities treated with different MIC concentrations of watercress oil loaded with Vaseline gel (OLG) and watercress oil only. Tube 2, 3, 4 treated with 25%, 50% and 75% MIC, respectively while tube 1 is the negative control (SDS), and tube 5 is the tested bacterium only (untreated). Anti-hemolytic activity for watercress oil and OLG in presence of *Klebsiella pneumoniae* **(A, B)**, *Escherichia coli* **(C, D)**, *Staphylococcus aureus* **(E, F)** and *Enterococcus faecalis* **(G, H)**.

It is well-known that certain bacteria produce toxins (virulence factor) with hematolytic effect due to their integration into the erythrocyte membranes, such as α-Toxin (Song et al., 1996). Therefore, the ability of watercress oil and OLG to inhibit/control the hemolytic effect caused by tested bacteria towards red blood cells was investigated. The inhibition of bacterial hemolytic activity by watercress oil and OLG was estimated when growing bacteria was treated with watercress oil and OLG, individually. Generally, it was evident that bacterial hemolysis was decreased with increasing quantities of MIC (%), ranging from 25% to 75% for both watercress oil and OLG. The rate of reduction/inhibition of bacterial hemolysis varied depending on tested bacterial species and used treatment products (watercress oil or OLG) (Figures 3, 4). Nevertheless, Vaseline gel has enhanced watercress oil for the inhibition of hemolysis produced by all tested bacteria at all tested concentrations. Interestingly, the hemolytic activity of tested *K. pneumoniae* was reduced by 8.0%, 4.4%, and 1.9% when using 25%, 50%, and 75% MIC of watercress oil alone, but a higher reduction rate (19.4%, 11.6%, and 6.8%) was achieved when OLG with the same MIC concentrations was applied, respectively. Hemolysis reduction was observed from 40.1% to 6.9%, 20.6%–4.7%, and 10.2%–1.7% when treated with 25%, 50%, and 75% MIC of watercress oil and OLG in the presence of *E. coli*, respectively.

Further detailed results regarding the inhibition of bacterial hemolysis (e.g., *S. aureus* and *E. faecalis*) are reported (Figure 3). Sodium Dodecyl Sulfate (SDS) was added to the suspension of erythrocytes as a positive control that showed a complete hemolysis in Eppendorf tube 5 (Figure 4). Watercress oil and OLG displayed no hemolytic potential as a control when tested without bacteria cells. This data could indicate the limited potential toxicity (hematolytic activity) of watercress oil and OLG towards red blood cells (RBCs) as well as their selective toxicity towards bacterial hemolytic toxins would lead to the suggestion that watercress oil and OLG would carry a great promising potential for therapeutic use in human health. Kim et al. reported the capacity of cinnamon bark oil to inhibit the biofilm formation of *Pseudomonas aeruginosa* resulting in low hemolytic activity. In another investigation, herring oil was reported to reduce the hemolytic influence of *S. aureus* against RBCs (Kim et al., 2018). Moreover, *S. aureus* is known to produce hemolysin that is destroyed by red blood cells which is a virulence factor that enhances the establishment of infection (Lee et al., 2022). The suppression/inhibition of hemolytic activity in the current investigation would highlight the importance of watercress oil and OLG for potential therapeutic implications to control this virulence factor (hemolysis) caused by all tested bacteria.

**FIGURE 5:**
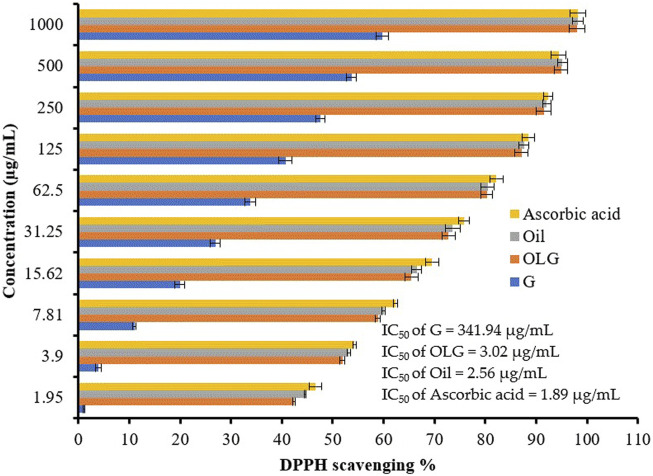
Antioxidant activity of watercress oil (Oil), watercress oil loaded with Vaseline gel (OLG), Vaseline gel (G), and ascorbic acid. Error bars in the figure represent the calculated standard deviation (SD). IC_50_ stands for Half-maximal inhibitory concentration.

Compared to ascorbic acid, a promising antioxidant activity was associated with watercress oil, which increases when increasing the concentration of watercress oil (Figure 5). However, Vaseline gel did not enhance the antioxidant activity of oil. This is because the current investigation on the antioxidant activities showed interesting value of IC_50_ (2.56 for watercress oil and 3.02 μL/mL for OLG). The lowest IC_50_ (1.89 μg/mL) was recorded for ascorbic acid. On the other hand, Vaseline gel alone showed low antioxidant activity with a high IC_50_ of 341.94 μg/mL. Watercress as a dietary supplement played an important role in minimizing the damage of DNA and inducing antioxidant action in the blood (Gill et al., 2007). The antioxidant activity of watercress oil may be attributed to phenolic compounds, such as coumaric acid and quercetin, as well as their derivatives, as previously mentioned by Zeb (2015). The different parts of the watercress, including leaves, roots, and stem, which reflected radical scavenging activity, were 81.6%, 78.0%, and 70.0%, respectively.

**FIGURE 6:**
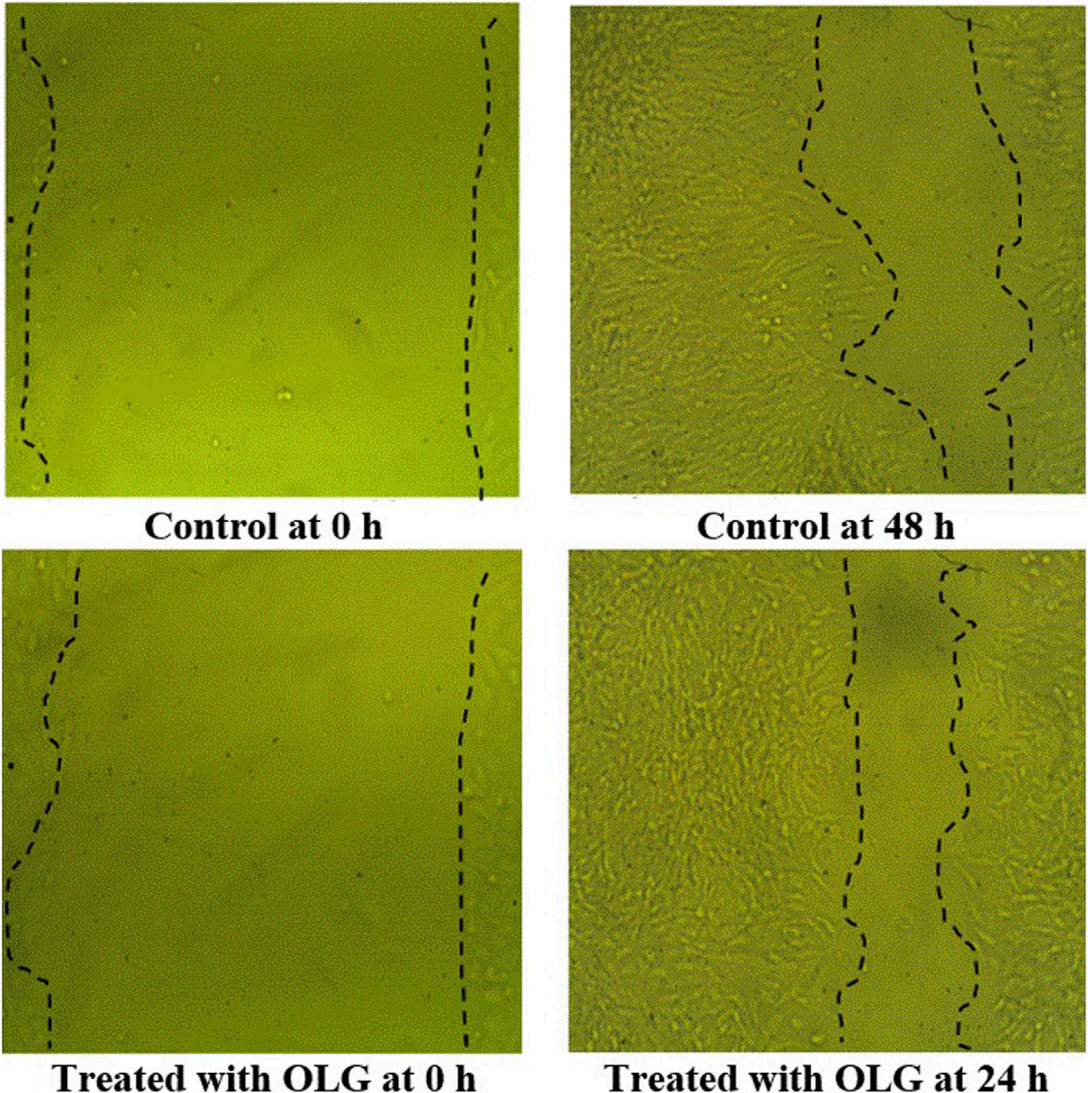
Scratch assessment assay to evaluate the influence of watercress oil loaded with Vaseline gel (OLG) towards cells on the area of wound at different time of exposures (0 and 48 h). Negative control (Untreated cells) was included for comparison.

OLG’s capability for the healing process was compared to untreated cells (Table 4; Figure 6) by measuring the cavity closer, which expresses the healing of wounds and the rapidity of the collective motion tissue. The visualized findings showed that all marks of healing via OLG, including migration rate, closure of wound, and difference of area (%), were 13.20 µm, 72.78 µm^2^, and 633.67%, compared with the untreated cells, which was monitored as 12.72 µm, 67.65 µm^2^, and 610.67%, respectively. These findings clarified that the oil formulated in gel form has succeeded in the wound’s healing. Minimizing tissue inflammation and oxidative stress may be represented as one of the healing mechanisms of OLG. Several investigators have formulated essential oils for wound healing acceleration; for instance, the formulated rosemary essential with nanostructured lipid promoted wound healing *in-vivo* by reducing bacterial colonization on tissues and minimizing the inflammation period (Khezri et al., 2019). Lemon grass oil formulated with Carbopol 940 gels showed the greatest antibacterial activity compared with bulk oil (Shukr and Metwally, 2013). Another oil was applied in topical form, namely, coconut oil, and encouraged the production of collagen and epithelialization process; hence accelerating the healing process of the wound and minimizing the healing time *in-vivo* (Hamza, 2012; Istek et al., 2023). Wilson et al. mentioned that preventing microbial infection and reducing prolonged inflammation are the strategies by which essential oils facilitate the process of wound healing (Wilson et al., 2015). According to the study conducted by Nour et al. (2023), cinnamon oil-loaded hydrogel revealed a potential efficacy for the management of methicillin-resistant *S. aureus* in infected wounds and enhanced the process of skin healing (Nour et al., 2023).

**TABLE 4:** Healing activity of the watercress oil loaded with Vaseline gel (OLG).

Treatment	At 0 h		At 24 h		At 48 h		RM um	Wound closure % um	Area difference %
	Area	Width	Area	Width	Area	Width			
Control (without treatment)	945	944.00	733	732.01	271	270.07	12.72	67.65	610.67
	923	922.04	673	672.19	363	362.05			
	897	896.02	627	626.05	363	362.02			
	881	880.04	487	486.037	177	176.41			
	869	868.01	611	610.003	247	246			
	901	900.01	535	534.015	331	330.10			
	Mean								
	902.67	901.69	611	610.05	292	291.11			
OLG	849	848.24	653	652.05	337	336.02	13.20	72.78	633.67
	805	804.02	609	608.03	255	254			
	817	816.25	507	506.06	209	208.01			
	937	936.03	537	536.09	157	156.05			
	879	878.02	463	462.21	187	186.10			
	937	936.05	529	528.06	277	276.03			
	Mean								
	870.67	869.77	549.67	548.75	237	236.04			

Overall, all observed promising biological activities (antibacterial, antifungal, and anti-hemolytic) of OLG and its great enhancement for wound healing could be somehow liked with its hydrophobic property. Although, all these data would suggest a great and promise therapeutic potential for OLG in human health, further investigations still required in relation to the identification of the exact/actual compound/s responsible for each activity, mode of action by which each activity was achieved and both *in-vitro* and *in-vivo* toxicities of the active compound/s and its formulation/s for each potential therapeutic use in human health.

## 4 Conclusion

The combination of watercress oil with Vaseline gel (OLG) has shown an increased efficacy against pathogenic bacteria and fungi, as indicated by the formation of inhibition zones and a reduction in the MIC and MBC values. In addition, OLG possesses a stronger anti-hemolytic activity towards produced hemolysis by all tested bacteria compared to unloaded oil. Although tested biological activities of watercress oil were enhanced when loading watercress oil with Vaseline gel (OLG), unloaded oil showed higher antioxidant activity than OLG. Furthermore, OLG demonstrated promising effects in promoting the migration rate, wound closure percentage, and area percentage compared to untreated cells, indicating its great potential use in wound care to enhance the process of wound healing. Therefore, the combination of watercress oil with Vaseline gel, as OLG, may offer a multifaceted approach to addressing microbial infections and promoting wound healing.

## Data Availability

The original contributions presented in the study are included in the article/Supplementary Material, further inquiries can be directed to the corresponding author.
