# Supplementary material for: Watercress oil loaded with gel: evaluation of hemolysis inhibition, antioxidant, antimicrobial, and healing properties
Source: Front Pharmacol. 2024 Aug 23;15:1424369. doi: 10.3389/fphar.2024.1424369 (PMC11377900; doi:10.3389/fphar.2024.1424369)
Supplement: Supplementary file 1 [file DataSheet1.PDF]

Data File C:\CHEM32\1\DATA\PP 16-7-2023 2023-07-16 10-46-05\001-5001.D
Sample Name: STD

===

Acq. Operator	: SYSTEM	Seq. Line :	50
Acq. Instrument	: hplc -2	Location :	Vial 1
Injection Date	: 7/17/2023 7:37:17 AM	Inj :	1
		Inj Volume :	5.000 µl

Acq. Method : C:\CHEM32\1\DATA\PP 16-7-2023 2023-07-16 10-46-05\POLYPHENOL 2023.M
Last changed : 7/16/2023 10:46:08 AM by SYSTEM
Analysis Method : C:\CHEM32\1\DATA\PP 16-7-2023 2023-07-16 10-46-05\POLYPHENOL 2023.M (Sequence Method)
Last changed : 7/17/2023 10:41:53 AM by SYSTEM
(modified after loading)
Additional Info : Peak(s) manually integrated

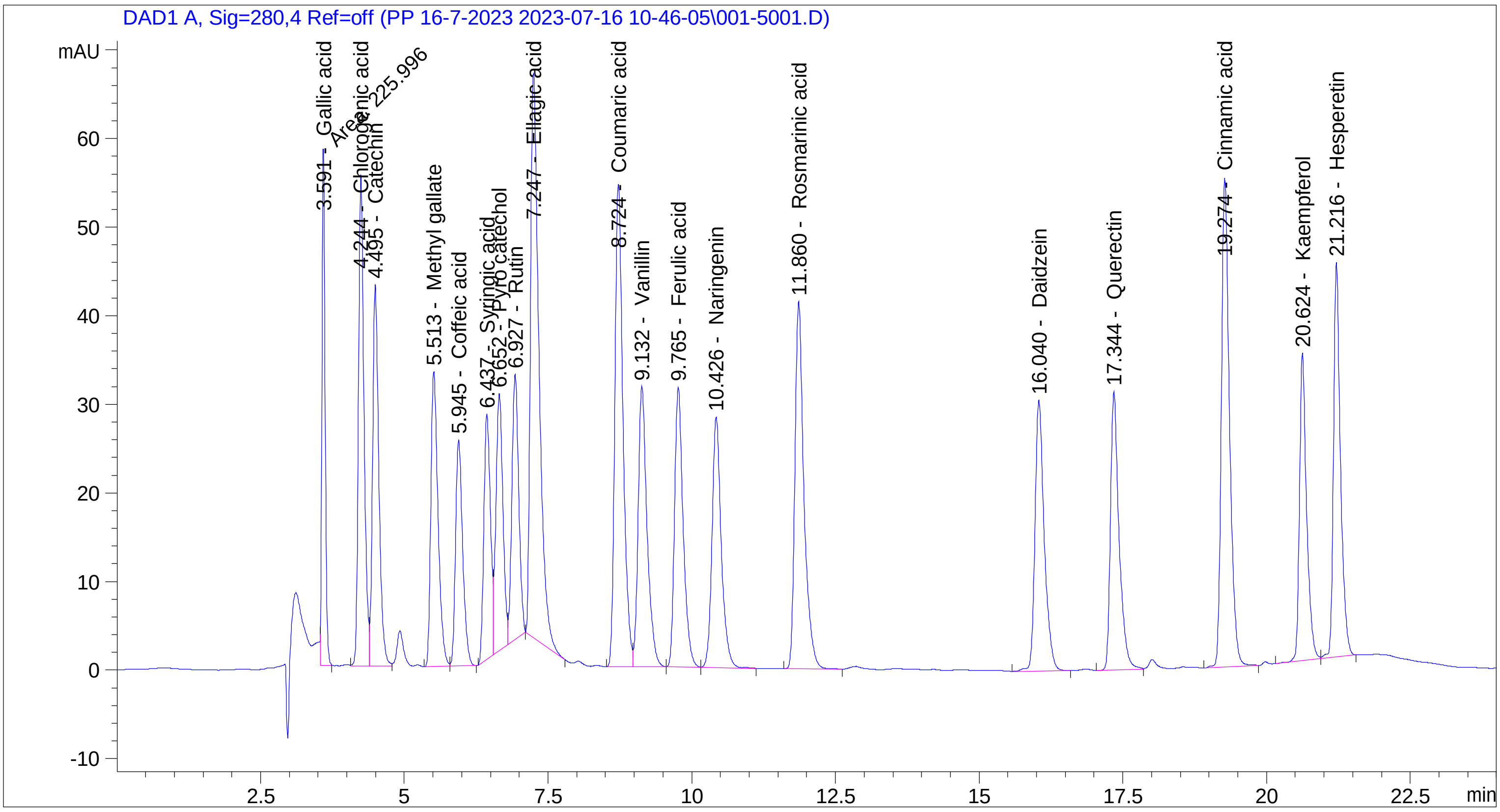

===
Area Percent Report
===

Sorted By : Signal
Calib. Data Modified : 7/17/2023 10:41:51 AM
Multiplier : 1.0000
Dilution : 1.0000
Use Multiplier & Dilution Factor with ISTDs

Signal 1: DAD1 A, Sig=280,4 Ref=off

Peak #	RetTime [min]	Type	Width [min]	Area [mAU*s]	Area %	Name
1	3.591	MM	0.0637	225.99588	3.3823	Gallic acid
2	4.244	VV	0.1022	372.85162	5.5802	Chlorogenic acid
3	4.495	VV	0.1144	326.41302	4.8852	Catechin
4	5.513	BV	0.1314	289.75479	4.3365	Methyl gallate
5	5.945	VB	0.1322	219.41479	3.2838	Coffeic acid

Peak #	RetTime [min]	Type	Width [min]	Area [mAU*s]	Area %	Name
6	6.437	BV	0.1227	219.94035	3.2917	Syringic acid
7	6.652	VV	0.1288	246.03615	3.6822	Pyro catechol
8	6.927	VB	0.1285	247.87781	3.7098	Rutin
9	7.247	BB	0.1576	663.69128	9.9330	Ellagic acid
10	8.724	BV	0.1493	538.87939	8.0650	Coumaric acid
11	9.132	VB	0.1596	341.38806	5.1093	Vanillin
12	9.765	BB	0.1571	328.00693	4.9090	Ferulic acid
13	10.426	BB	0.1657	314.32266	4.7042	Naringenin
14	11.860	BB	0.1640	455.12982	6.8116	Rosmarinic acid
15	16.040	BB	0.1669	343.63199	5.1429	Daidzein
16	17.344	BV	0.1533	327.05664	4.8948	Querectin
17	19.274	BB	0.1472	545.19739	8.1596	Cinnamic acid
18	20.624	BV	0.1263	299.10123	4.4764	Kaempferol
19	21.216	VB	0.1266	377.01865	5.6425	Hesperetin

Totals : 6681.70847

20 Warnings or Errors (10 first messages follow) :

Warning : Calibration warnings (see calibration table listing)
Warning : Invalid calibration curve, (Gallic acid)
Warning : Invalid calibration curve, (Chlorogenic acid)
Warning : Invalid calibration curve, (Catechin)
Warning : Invalid calibration curve, (Methyl gallate)
Warning : Invalid calibration curve, (Coffeic acid)
Warning : Invalid calibration curve, (Syringic acid)
Warning : Invalid calibration curve, (Pyro catechol)
Warning : Invalid calibration curve, (Rutin)
Warning : Invalid calibration curve, (Ellagic acid)

==

*** End of Report ***
